# Crystallinity and Oscillatory Shear Rheology of Polyethylene Blends

**DOI:** 10.3390/ma16196402

**Published:** 2023-09-26

**Authors:** Dorottya Nagy, Zoltán Weltsch

**Affiliations:** 1 Department of Innovative Vehicles and Materials, Faculty of Engineering and Computer Science, John von Neumann University, 10 Izsáki Street, 6000 Kecskemét, Hungary; nagy.dorottya@gamf.uni-neumann.hu; 2 Vehicle Industry Research Center, Széchenyi István University, Egyetem Tér 1, 9026 Győr, Hungary

**Keywords:** polyethylene, blend, rheology, crystallinity

## Abstract

Crystallinity and rheological behavior are significant for processing semi-crystalline polymers with fine mechanical properties. There is always an economical need to create a less expensive new material with better properties. Non-isothermal crystallization and oscillatory shear rheology of different branch-type polyethylene–polyethylene blends were investigated. Samples of high-density and low-density polyethylene (HDPE/LDPE) (20/80, 40/60, 60/40 and 80/20 weight ratios) and two types of high-density and linear low-density polyethylene (HDPE/LLDPE) (40/60 and 60/40 weight ratios) were prepared via extrusion. The materials were tested by differential scanning calorimetry (DSC) at several cooling rates (5, 10, 20, 30 and 40°/min) and by oscillation rheometry (ARES G2) at low angular frequency range to prove their miscibility or immiscibility. It was found that the one-peak melting endotherm of the 80–20% HDPE-LDPE blend could indicate miscibility in the solid phase, while the other HDPE-LDPE blends with two-peak curves are partially or not miscible. In contrast, all the HDPE-LLDPE blends indicate co-crystallization, but the 40–60% HDPE-LLDPE butylene blend is probably immiscible. It was revealed that complex viscosity decreases with angular frequency: linearly for HD-LD blends and not linearly for HD-LLDPE blends. The complex viscosity shows linear behavior with composition for HD-LLDPE blends, while there is a positive–negative deviation for HD-LD blends. In the liquid phase, according to rheological measurements, the HDPE-LDPE blends are not or partially miscible, while the HDPE-LLDPE blends are probably miscible.

## 1. Introduction

Polyethylene (PE) has been widely used in the packaging industry for more than 80 years. The structure of PE is simple; however, the content and length of the side chain branching determine the degree of crystallinity, and the mechanical properties [1]. According to the branch content and density, there are several types of PE: high density (HDPE), low density (LDPE), linear low density (LLDPE), ultrahigh molecular weight (UHMWPE), etc. [2]. There is always an economical need to create a less expensive new material with better properties, so there are continuous studies on blends to combine the material properties [3,4,5,6,7].

Using polyethylene–polyethylene blends, the thermal and mechanical properties change with the branching degree. The final material behavior will be composed of the molecular weight (MW), molecular weight distribution (MWD) and the number of the short- and long-chain branches of the original materials [8].

Agrawal et al. investigated the melt miscibility of HDPE/LDPE and HDPE/LLDPE blends and found that the HDPE/LLDPE blends are miscible, while the HDPE/LDPE blends are immiscible because of the long-chain branching of LDPE (LCB). The relaxation times of hexene comonomer containing LLDPE blends were higher than butane [9].

On the other hand, LCB can enhance the shear thinning behavior, which can be beneficial for blow molding or film preparation [10]. The LCB containing m-HDPE is miscible in m-LLDPE and with the HDPE content the melting temperature increases.

Several researchers investigated the effect of MW on miscibility. Some said that the effect of MW on miscibility can be neglected [11,12]. Others said that materials with lower MW are more miscible [13,14].

Hameed et al. (2006) found that in the solid phase, the miscibility is independent of the molar mass or the branch degree. However in the liquid phase, the HDPE/LLDPE blend with lower MW is miscible, but with higher MW there is a negative deviation with log additivity rule, which could be caused by layered morphology. The HDPE-rich phase is compatible because co-crystallization has occurred. In the LLDPE-rich phase, several exotherm peaks by DSC show separated crystallites [15].

When mixing the HDPE-LDPE mixture, there is a contradiction in terms of composition. In the study of [5,16], mixing was possible at low HDPE content, as the distribution of HDPE crystals in the LDPE matrix improved. In the research of [7,11], however, the blend with a high HDPE content was mixed.

In the HDPE-LDPE mixture, phase separation often occurs in the crystalline phase [1,11,17,18,19,20,21,22,23,24], since crystals of different sizes melt at different temperatures and those with different structures and materials create different crystal modifications. That is why the slower the blend is crystallized by cooling, the more the differences between the crystal structures appear, and the more it segregates [24,25]. This also means that rapid cooling can prevent segregation [26]. If the raw materials are not mixed, the components still interact, and a fractionation effect occurs [27,28]. This means that parts of the raw materials with similar branching/density act on each other as nucleators, so they crystallize at the same time.

Based on experiments, HDPE/LLDPE blends mix better with a high HDPE content [15,29].

Samples with broad MWD values show higher Newtonian viscosity, which could be useful in manifesting long-chain branching [30]. Long-chain branches can cause phase separation, and make the material thermorheologically complex; however, this field is quasi-unknown for Arrhenius-type polymers [31,32,33].

Hussein et al. also predicted that the increasing branch degree makes the polymer melt more immiscible. However, LLDPE/LDPE blends with high hexene comonomer content are miscible, while those with low hexane comonomer content are immiscible [34].

Chen et al. measured ultrahigh molecular weight polyethylene (UHMWPE) and the blends with LDPE were miscible, while with LLDPE, the miscibility had strong weight fraction dependence and micro-phase separation occurred. The measured three endotherm peaks by differential scanning calorimetry (DSC) show co-crystallization. The crystallization rate of the pure materials differs, so liquid–solid phase separation forms. That also indicates a decrease in crystallization temperature [35].

Some investigated the effect of catalyst type (Ziegler–Natta (ZN) or metallocene (m)).

Hill et al. found that there is no effect of the catalyst type [36]. However, Lee et al. measure the ZN-LLDPE to be more miscible in HDPE, than m-LLDPE [7]. Hussein et al. found that in the m-LLDPE-rich phase, the other polymer with high BC is less miscible [34].

Melt miscibility in the function of branching structure can be investigated via time–temperature superposition (TTS), Han plot (G’ vs. G”), van Gurp-Palmen plot (δ vs. G*), Cole–Cole plot (η” vs. η’) and viscosity vs. concentration (blend ratio) curve [9,10,16,35,37,38,39].

The crystalline phase can be investigated by calorimetry. The cooling rate influences the quantity and structure of crystals. In blends, generally, a co-crystallized phase improves the properties [40,41]. The co-crystallization is a sign that the blend is compatible with the crystalline phase [42,43]. In some cases, more than one type of crystalline structure is formed [4]. The fact that phase separation or co-crystallization is generated is a matter of blend ratio [15]. By calorimetric investigation, one melting peak can indicate complete co-crystallization, while two melting peaks might indicate phase separation [1]. The mechanical properties of both the phase-separated and the co-crystallized blends can be better, compared with the original PE’s [42].

During crystallization, we can see that the blends do not mix [44,45], or only separate due to slow cooling [46]. But they can also mix [6,7,27,40,47] and co-crystals are formed even with slow cooling [tashiro1999] or partial melting in the melt state [48].

Usually, processing of the thermoplastics happens in a molten state (extrusion, injection molding), so knowing the rheological properties is essential. The molar mass influences the melt strength and the viscosity [1], the melt strength is also affected by the branching structure: for LDPE the highest, then for HDPE, and for LLDPE the lowest [49]. Some researchers said that not just the branch content, but also the branch distribution determine the properties [50]. The melt miscibility is not affected by the high viscosity difference of the base materials [51,52,53,54].

The rheological investigations of polyethylene blends are usually between 180 and 210 °C; however, there are PE types, which are suggested to prepare even at 270 °C. Recently, several authors investigated HDPE-LDPE or HDPE-LLDPE blends [5,15,16,29,34,38,42,51,55,56,57,58]; however, only a few deal with the comparative study of these blends at elevated temperatures (230–250 °C).

Therefore, in this study, blends of high-density and low-density polyethylene (HDPE/LDPE) (20/80, 40/60, 60/40 and 80/20 weight ratios) and two types of high-density and linear low-density polyethylene (HDPE/LLDPE) (40/60 and 60/40 weight ratios) were prepared via extrusion. Results of non-isothermal crystallization and oscillatory shear rheology of different comonomer-type polyethylene–polyethylene blends are shown.

## 2. Materials and Methods

HDPE TIPELIN FA 381-10 (with hexen-1 comonomer) [59], LDPE TIPOLEN FB 243-51 (not containing any additives) [60] and two types of LLDPEs: Exceed 3518 (with hexane comonomer) [61] and Flexirine CL10U (with butylene comonomer) [62] were selected. HDPE and LDPE are produced by MOL Group, TVK Nyrt. Hungary. LLDPEs are produced by Exxon Mobile Chemicals and ENI Versalis, respectively. The Melt Flow Rate of the four polyethylene types was determined at 190 °C with 2.16 kg. The MFR values are 0.28, 0.75, 3.5 and 2.5 g/10 min, respectively. Their densities at 23 °C are 937, 921, 918 and 918 kg/m^3^, respectively (Table 1).

The PE-PE blends were made by a Collin Teach-line conventional extrusion line with an E20T single-screw extruder, with 190–200–210 °C zone temperatures and 80 rpm extruder revolution. The feed rate of the granulate in the extruder is 0.7 kg/h. These standard parameters were used to make recycled samples along the blends by producing filament from the original materials and grinding them. Taking everything into consideration, the starting material was the same in the recycled case, only the ones produced along the mixtures were processed once by a grinding process after the production of filament and then extruded once more. Recycled samples are useful, because they contain the same mechanical and heat impact as the blends, so they are ideal for references. The original materials mean the samples obtained from the manufacturers. They were measured without preprocessing (extrusion).

HDPE/LDPE and two types of HDPE/LLDPE blends were made in 100/0, 80/20, 60/40, 40/60, 20/80, 0/100 and 100/0, 60/40, 40/60, 0/100 weight ratios, respectively. In the notation of the blends, the first number is always the HDPE percentage and the second number is the other PE (abbreviated as LD, Flex or Ex) percentage.

Melting and crystallinity of the samples were investigated by differential scanning calorimetry (DSC). Thermal Analysis TA Q200 heat-flux DSC equipment was applied according to the ISO-11357-1 standard [63]. All the samples were between 3 and 5 mg. The measuring atmosphere was nitrogen gas with a 50 mL/min flow rate. At non-isothermal conditions (between 30 and 180 °C), the cooling rate was 5, 10, 20, 30 and 40 °C/min.

The rheological behaviors of the samples were characterized by oscillatory shear rheometry ARES G2 with an SMT system (Separated Motor Transducer) and with an FCO (Forced Convention Oven) [64]. A 25 mm diameter cone-plate geometry was applied and approximately 0.5 g granulate was used for each measurement. The angular frequency was between 6.28 and 628 rad/s at 0.1% strain. The trim gap was 0.09 mm and the geometry gap was 0.04 mm. The HDPE/LDPE blends were investigated at 230 °C, while the HDPE/LLDPE blends at 250 °C. These temperatures can seem high compared to the processing temperature of common polyethylenes, but both LLDPEs require a higher processing temperature according to the manufacturers. For example, it is around 270 °C for the Exceed LLDPE [61]. For the HDPE/LDPE blends we tried to find a temperature near the other measuring temperature without too high flowability.

## 3. Results and Discussion

DSC measurement is appropriate to investigate the chemical or physical changes in polymers such as crystal melting or crystallization. The first melting curve can show information from the processing, so the second melting curve was investigated.

The melting endotherms of the HDPE-LDPE blends show a two-peak curve. The peak temperature of the recycled materials is higher than the originals, and the difference is increasing with the heating rate (~3 °C at 40 °C/min). The melting endotherm of the recycled materials is also broader and increases with the heating rate. The butylene and hexane LLDPEs show two peaks melting at 5 °C/min, and the two peaks merge into one with the heating rate. Before the melting peaks, all the materials and blends have a subpeak at 110 °C, which does not change with the rate. It is possible that the more branched parts melt at lower temperatures. All the HDPE-LLDPE blends have a one-peak melting, which can indicate co-crystallization. However, the peak temperature of the 60–40% HDPE-LLDPE butylene blend and all the HDPE-LLDPE hexane blends are between the original materials and the height of the peak is proportional to the HDPE content, the peak temperature of the 40–60% HDPE-LLDPE butylene blend is near to the peak temperature of HDPE and the height of the peak is smaller by a lot.

Shen et al. found that during the crystallization of mixtures, a peak is visible on DSC, which may indicate co-crystallization (linear fraction of HDPE and LLDPE co-crystallize) or overlapping of peaks (HDPE acts as a nucleator for LLDPE, while LLDPE acts as a diluent for HDPE- re, so the vertices meet). Due to the SCB content, the chain regularity of LLDPE is lower, therefore a smaller, wider, lower temperature peak is formed. By increasing the HDPE component, the area of thicker lamellae (at higher temperatures) shows a positive difference, as more perfect crystals are formed. According to this, the degree of crystallinity is proportional to the HDPE content and this affects the melting and crystallization temperature as well [42].

Based on Hameed et al.’s research, the compatibility in the solid phase of low molecular weight HDPE/LDPE blends is independent of molecular weight and branching but depends on the HDPE content (the more content, the more co-crystals) [15].

Hill et al. also found for linear and branched PE blends that segregation occurs at low linear PE contents. Co-crystallization occurred at a ratio of 6:4 (linear-branched) [11].

**Figure 1:**
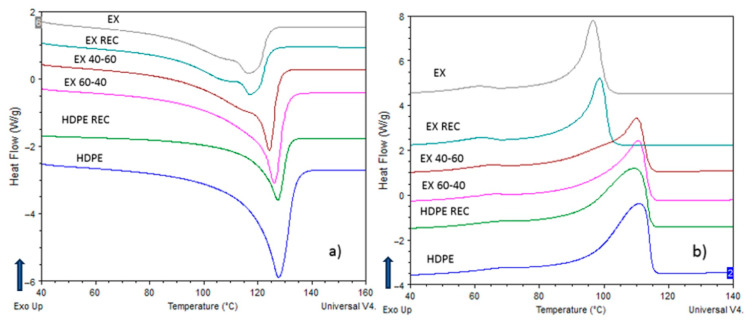
Melting (**a**) and crystallization (**b**) curves of HDPE-LLDPE (Exceed) blends at 20 °C/min heating/cooling rate.

Melting and crystallization curves of HDPE-LLDPE (Exceed) blend at 20 °C/min heating and cooling rate can be seen in Figure 1. The crystallization process starts at a higher temperature for the blends, while for the original and recycled LLDPE (EX) it is more than 10 °C later. All the materials have a postcrystallization at around 65 °C (for HDPE-containing materials it is a higher value), so the blends have a broader crystallization interval.

The double-peaked melting endotherm and crystallization exotherm seen in the HDPE-LDPE mixtures and the single-peaked melting endotherm and crystallization exotherm seen in the HDPE-LLDPE mixtures (Figure 1) indicate that in the former case, miscibility depends on the composition and partial miscibility is possible, while in the latter, co-crystallization occurs and the blends are miscible (besides the 40–60% HDPE-LLDPE butylene blend).

**Figure 2:**
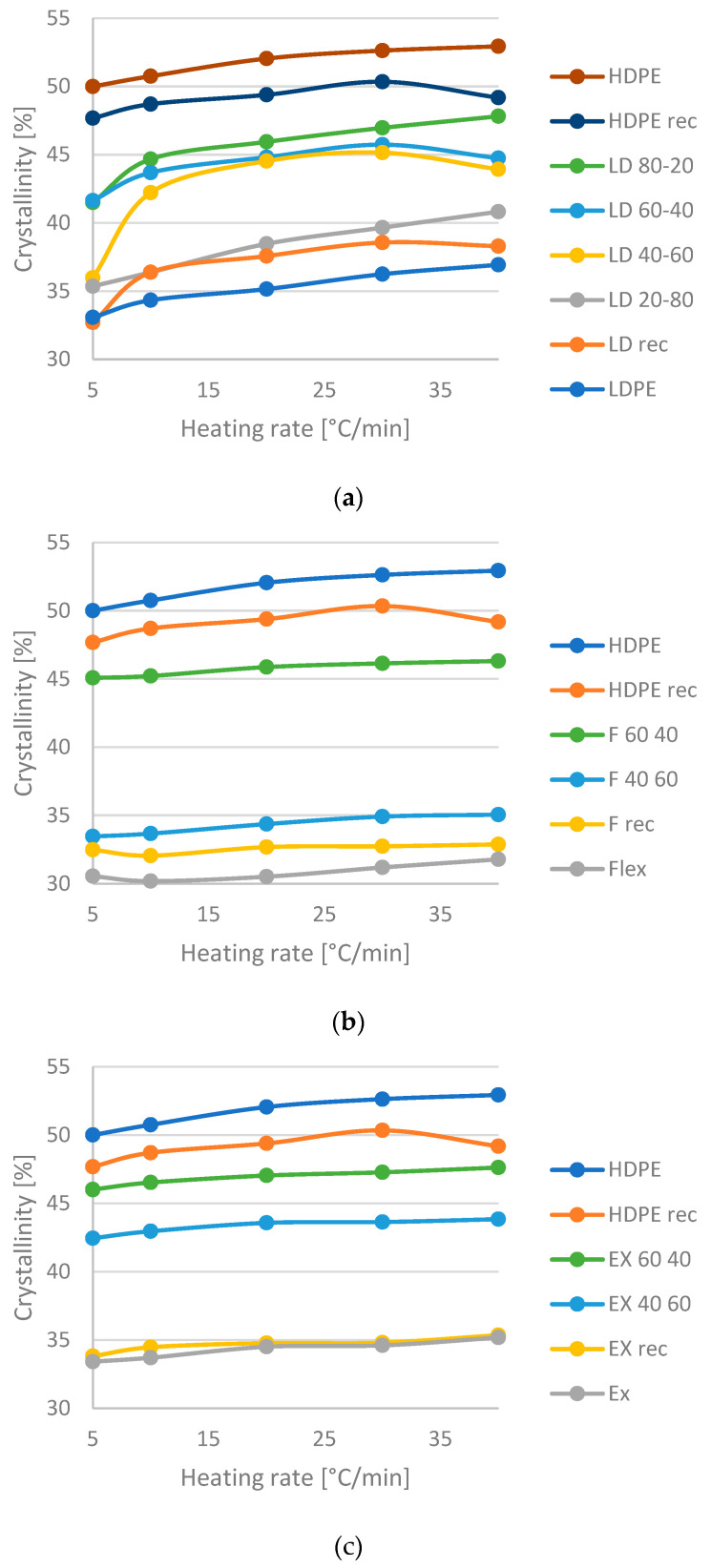
Crystallinity in the function of heating rate: (**a**) HD-LD blends, (**b**) HD-Flex blends and (**c**) HD-Ex blends.

Crystallinity is shown in the function of the heating rate (Figure 2) All three blends show crystallinity increasing with the heating rate; however, in Figure 2a at 5 and 40 °C/min some crystallinity values show deviation. This could be caused by phase separation. The HDPE has larger, more uniform crystallites, so the crystallinity is escalating with HDPE content. As the HDPE content increases, phase inversion occurs, and the matrix and dispersed phase switch place, HDPE will be the matrix and the other PE will be the dispersed phase. The heating and cooling rate influence the crystallization process. The lower rate enhances the phase separation [25], while a much higher rate can hinder it [26]. The location of the original and recycled materials is different for each PE. The processing can cause damage to the linear polymer chain, so the recycled HDPE has lower crystallinity. Recycled Flex and recycled LDPE have a higher value, while the value of recycled Ex does not change. The processing does not have the same effect on the small chain branches as the longer chains. In all cases, the crystallinity of blends is between the base materials and the recycled ones. For HD-LD blends (Figure 2a), between 10 and 30 °C/min and from 40 to 80 wt% HDPE content, the degree of crystallinity seems similar to the expected value of 60–40% blend. A characteristic crystalline structure is probably formed at these velocities.

The possible phase separation could be between 20 and 40 wt% HDPE content. For HD-Flex blends (Figure 2b), there is a gap between 40 and 60% HDPE content, which can indicate phase separation or alteration. For HD-Ex blends (Figure 2c), all the curves are nearer to the HDPE curve, than to the Ex curve. This means that, if there is phase separation, it is under 40% HDPE content. Comparing the curves with the ones calculated by the mixing rule, the 40–60% HDPE-Flex blend is falling behind, while the 60–40% blend is better than the calculated. All the HDPE-Ex blends are better than expected.

**Figure 3:**
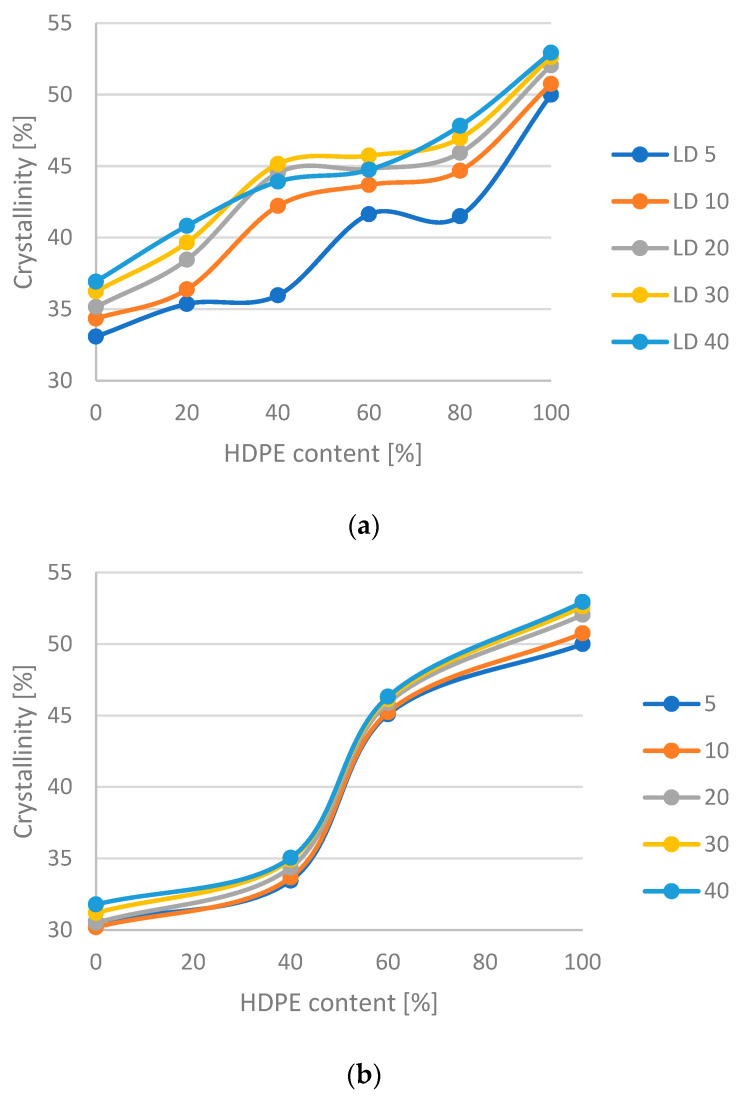

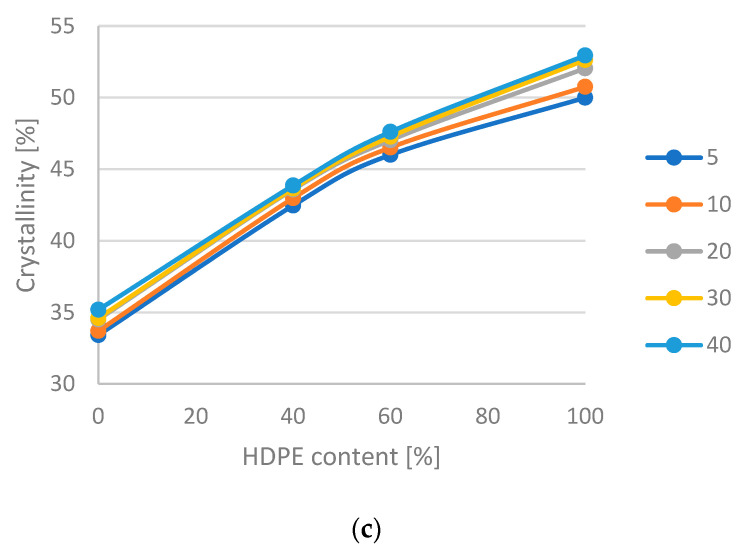
Crystallinity in the function of HDPE content: (**a**) HD-LD blends, (**b**) HD-Flex blends and (**c**) HD-Ex blends.

In Figure 3a,b, the crystallization shows a large deviation from linearity, which indicates that the mixtures are not miscible in all compositions, while in Figure 3c there is only a small positive deviation, which may mean that the mixtures are partially or completely miscible. The heating rate only has an effect on the degree of crystallization of HDPE-LDPE mixtures, even there only at very low (5 °C/min) and very high (40 °C/min) rates, so this is probably due to the uncertainty of the equipment at 40 °C/min. At 5 °C/min, the degree of crystallinity follows the decreasing trend seen at the other heating rates, but phase transitions occur at other compositions. It is possible that at 5 °C/min the structure changes between 40 and 60% HDPE content, while at higher speeds between 20 and 40%. It is likely that the in Figure 3b phase separation occurs between 40 and 60% HDPE content.

**Figure 4:**
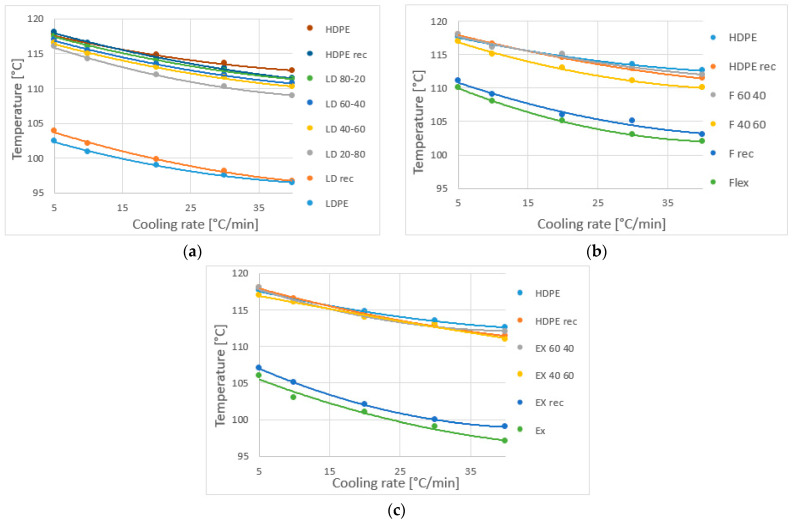
The starting temperature of crystallization as a function of cooling rate: (**a**) HD-LD blends, (**b**) HD-Flex blends and (**c**) HD-Ex blends.

The extrapolated starting temperature of crystallization is shown as a function of the cooling rate (Figure 4). The starting temperature decreases with the cooling rate. While HDPE fractions form crystallites at higher temperatures (because of the linear structure), the starting point of crystallization of blends is significant for HDPE. Therefore, all the blends have a similar starting temperature of crystallization. The processing does not affect all the long chains, so recycled HDPE and original HDPE diagrams are the same, except at 40 °C/min, where there is too little time to form the same crystallite. Nevertheless, for the LD, Flex and Ex materials, the recycled ones have higher starting temperatures. Maybe after extrusion, the chains remained oriented.

**Figure 5:**
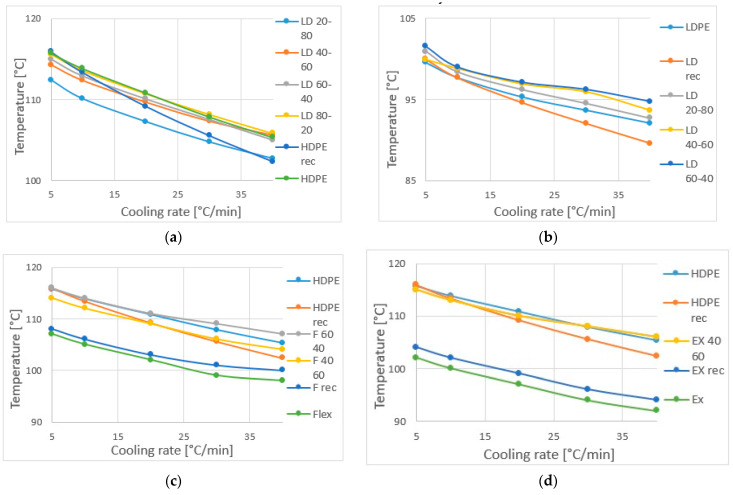
Peak temperature of crystallization as a function of cooling rate: (**a**) HD-LD blends first peak, (**b**) HD-LD blends second peak, (**c**) HD-Flex blends and (**d**) HD-Ex blends.

The peak temperature of crystallization decreases with the cooling rate (Figure 5). HD-LD blends (Figure 5a,b) have two peaks, where the first peak (at higher temperatures) is specific for HDPE and the second peak is specific for LDPE fraction. Until 60% LDPE content, the first peak temperature is not shifted by the other ingredient, and the second peak temperature is shifted in little extent only at a higher cooling rate. The peak temperature curve of the recycled materials is always steeper than that of the originals or the blends, so the cooling rate influences the peak temperature more for these recycled materials. In contrast, for Flex and Ex (Figure 5c,d) the curve steepness of the original and the recycled materials are the same. For the LLDPE blends, the peak is determined mainly by HDPE. The maximum temperature of Ex was lower than that of Flex; however, Ex affected the peak temperature less. The recycled LLDPE materials (Flex and Ex) has slightly higher peak temperature than the originals.

The rheological behavior can be characterized by complex viscosity and complex modulus values investigated by shear oscillation.

**Figure 6:**
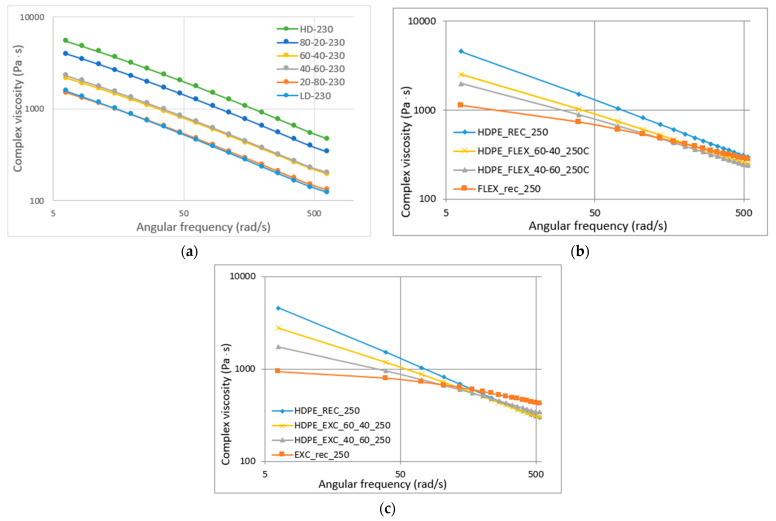
Complex viscosity in function of angular frequency: (**a**) HD-LD blends, (**b**) HD-Flex blends and (**c**) HD-Ex blends.

The complex viscosity is represented in the function of angular frequency, both axes use the logarithmic scale (Figure 6). In general, complex viscosity decreases with angular frequency. For HD-LD blends (Figure 6a), the curves are parallel, so the viscosity changes the same way for all the materials. LDPE and the 20–80% blends; and the 40–60% and 60–40% blends have the same viscosity values at all frequencies, respectively. Even a small percentage of LDPE could decrease the viscosity of HDPE. For LLDPE blends (Figure 6b,c), the curves are not parallel, at lower frequencies there are bigger differences because the complex viscosity is more sensitive to the structure [9]. At higher values, the curves are intersecting. For HD-Ex blends (Figure 6c), this intersection is more regular than that of HD-Flex blends. Cho et al. found the same curve shapes for HDPE-LDPE (parallel) and HDPE-LLDPE (intersecting) [1]. They concluded that all the blends were miscible, but the complex viscosity of their blends showed linear behavior with composition.

**Figure 7:**
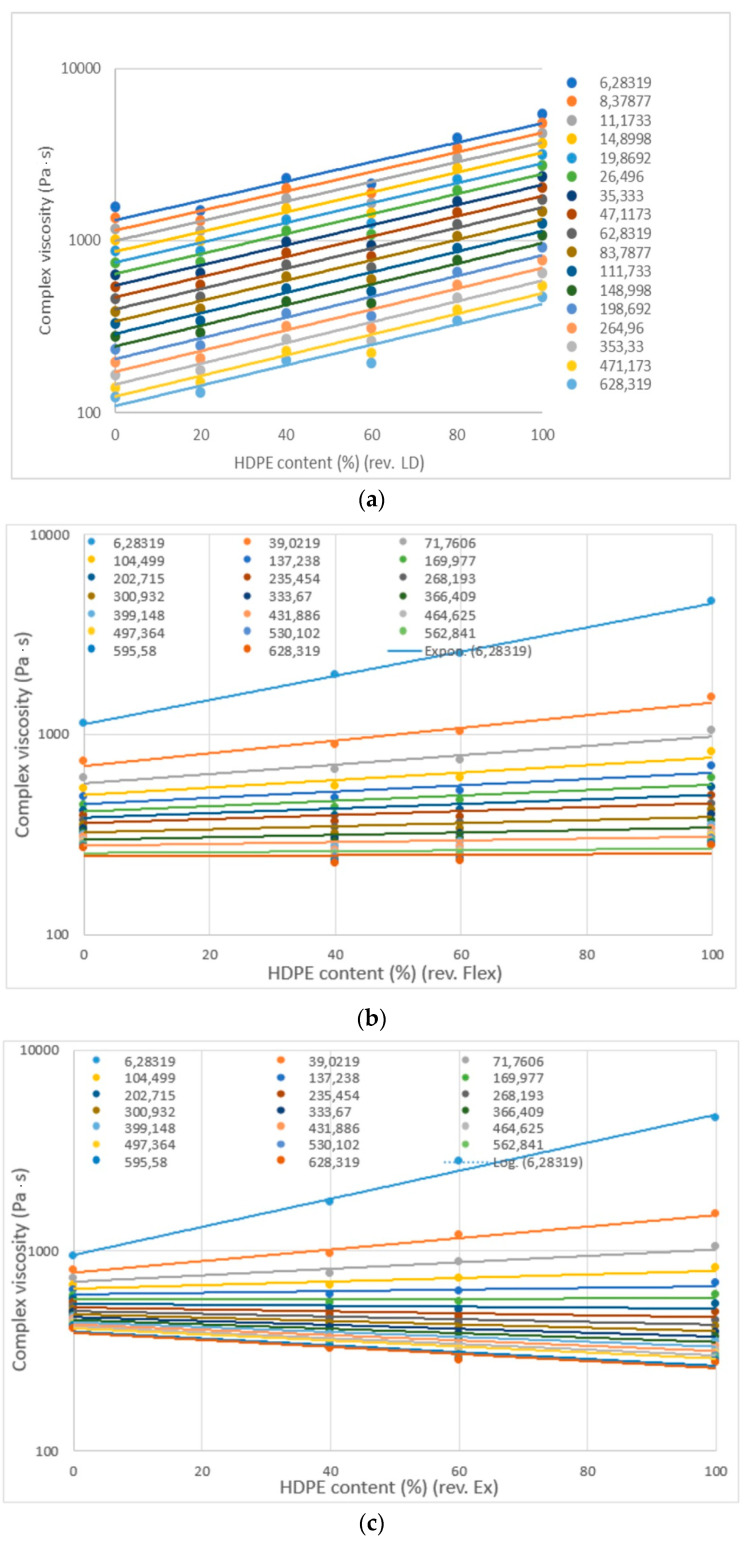
Complex viscosity in function of HDPE content: (**a**) HD-LD blends, (**b**) HD-Flex blends and (**c**) HD-Ex blends.

Plots of complex viscosity versus HDPE content show a linear relationship; however, 20–80% and 60–40% HDPE-LDPE blends have a significant negative deviation, while HDPE-LLDPE blends have a smaller negative deviation. Curves where the angular frequency difference was equal (Figure 7b,c) show that at lower frequency values complex viscosity is influenced more by angular frequency.

**Figure 8:**
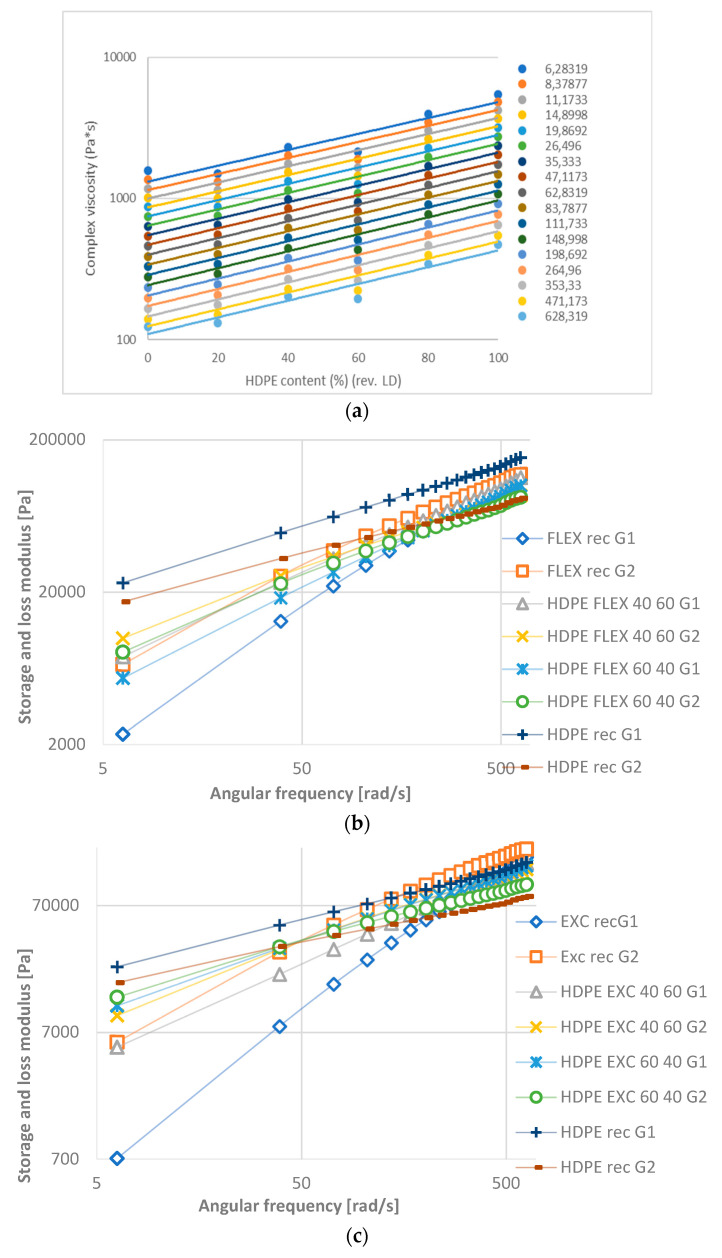
Storage and loss modulus in function of angular frequency: (**a**) HD-LD blends, (**b**) HD-Flex blends and (**c**) HD-Ex blends.

The complex modulus contains the storage and loss modulus parts, which increase in function of angular frequency (Figure 8). The storage modulus curves (G1) of HD-LD blends are parallel, while those of HD-LLDPEs have intersections. LDPE and the 20–80% blends and the 40–60% and 60–40% blends have the same modulus values at all frequencies, respectively (Figure 8a). The loss modulus curve of HD-Ex blends is the most orderly, the curves intersect at one point, at 50 rad/s.

The given storage (G1) and loss (G2) modulus curves have an intersection in the measured interval (despite HDPE, Flex and Ex recycled materials), which show the polydispersity of the samples. For HDPE-LDPE blends ((Figure 8a), PDI increases with HDPE content.

**Figure 9:**
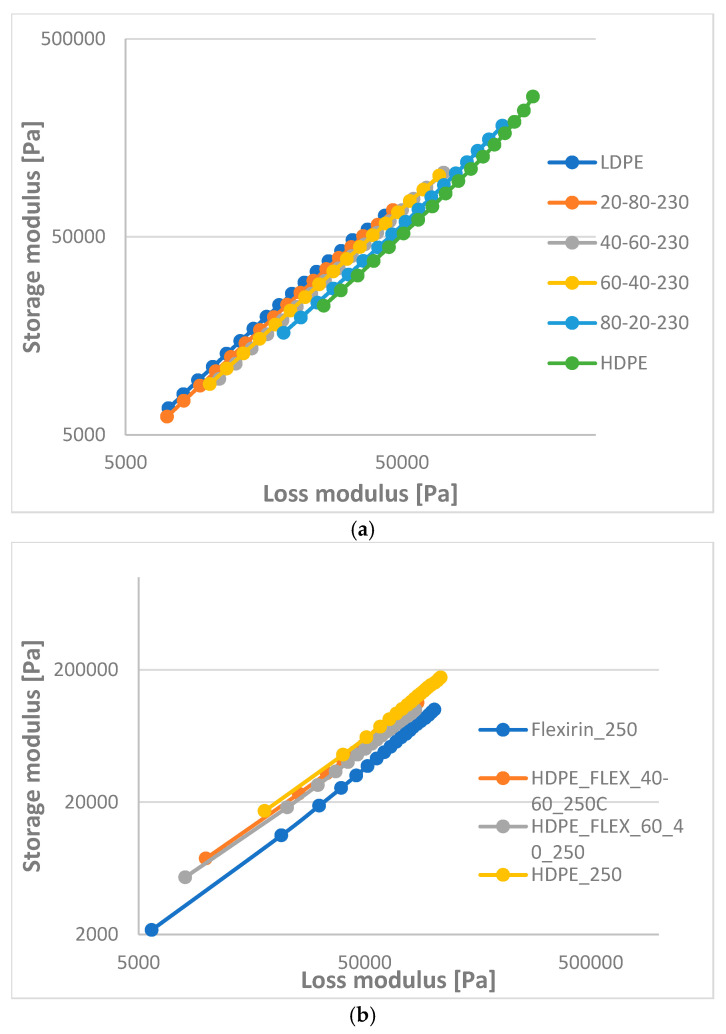

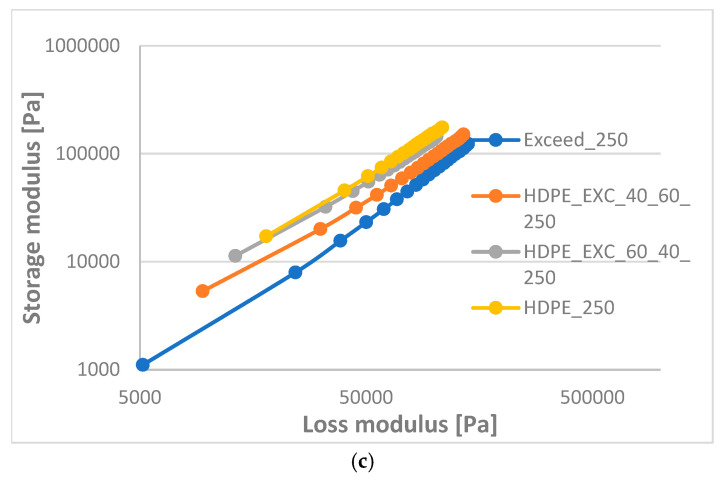
Han plot of (**a**) HD-LD blends, (**b**) HD-Flex blends and (**c**) HD-Ex blends.

In Figure 9, Han plots are shown, (a) for HDPE-LDPE blends at 230 °C, (b) for HDPE-LLDPE (Flex) blends at 250 °C and (c) for HDPE-LLDPE (Ex) blends at 250 °C. Han plots (log storage modulus (G’) versus log loss modulus (G”)) are independent of composition or temperature for compatible blends. However, none of the blends are independent of composition. HDPE-LDPE blends show a good similarity to LDPE or HDPE, which means that one or more blends are compatible, but certainly, there is a phase separation at a certain composition. A 20–80% blend is similar to LDPE, 40–60% and 60–40% blends have the same values between the original materials, while an 80–20% blend is similar to HDPE. Curves of the HDPE-LLDPE (Flex) blends are between the original materials and are similar to HDPE. A 60–40% blend of HDPE-LLDPE (Ex) can be compatible because its curve is on the HDPE curve. The 40–60% blend is probably immiscible.

**Figure 10:**
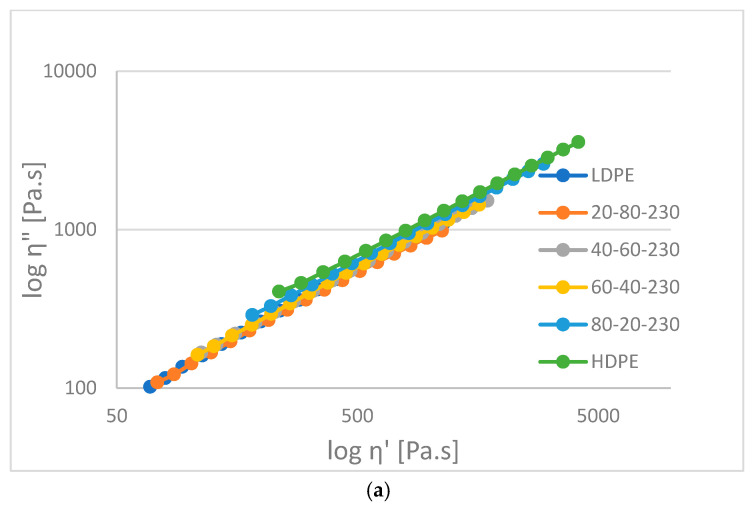

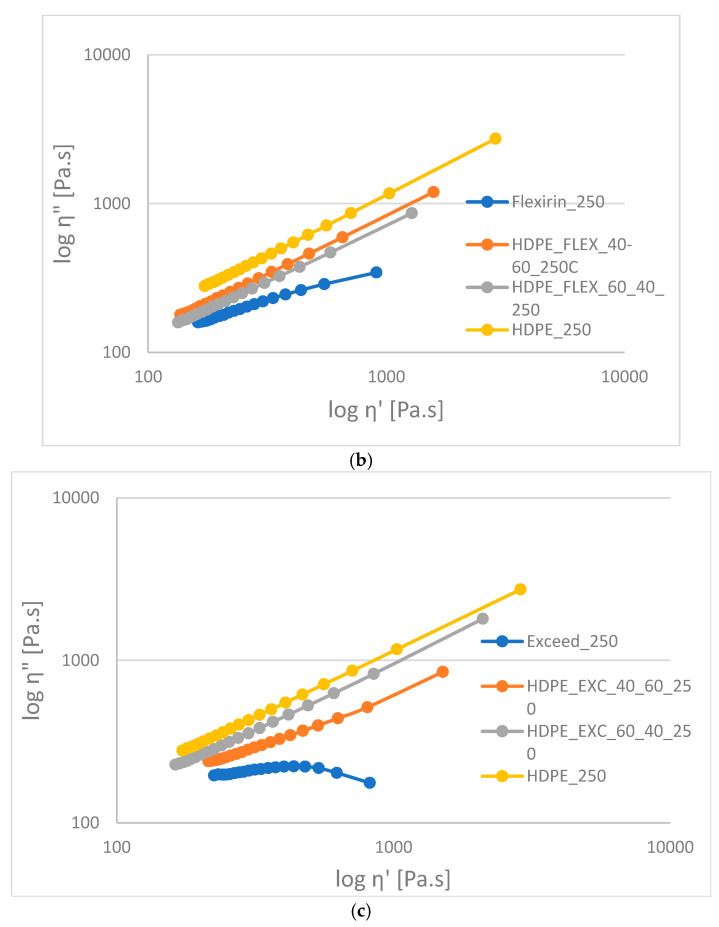
Cole–Cole plots for (**a**) HD-LD blends, (**b**) HD-Flex blends and (**c**) HD-Ex blends at 250 °C.

In Figure 10, the imaginary part of viscosity versus the real part of viscosity (Cole–Cole plot) are shown for (a) HDPE-LDPE, (b)HDPE-LLDPE (Flex) and (c) HDPE-LLDPE (Ex) blends. In the case of miscible blends the Cole–Cole plot forms a parabola at a higher loss viscosity range (at a lower frequency). In the measured region, the elastic–viscous behavior can be seen. The HDPE-LDPE blends are similar to each other; however, the more HDPE content, the more viscous are the blends. The HDPE-LLDPE (Flex) blends are similar to each other but are between the original materials. The 40–60% blend is more viscous than the 60–40% blend. On the other hand, the slope of the HDPE-LLDPE (Ex) blends are different. The 60–40% blend is more viscous and similarly as good as HDPE. At this frequency region, it is not sure that the blends are immiscible.

## 4. Conclusions

Three types of polyethylene–polyethylene blends were processed via extrusion and characterized by calorimetry and rheometry. This study concluded the following:

- The one-peak melting endotherm of the 80–20% HDPE-LDPE blend could indicate miscibility in the solid phase, while the other HDPE-LDPE blends with two-peak curves are partially or not miscible.
- All the HDPE-LLDPE blends have a one-peak melting, which can indicate co-crystallization. However, according to the peak temperature and the height of the peak (degree of crystallinity) of the 40–60% HDPE-LLDPE butylene blend is immiscible, while all the other HDPE-LLDPE blends are miscible in solid phase.
- According to the change in crystallinity, the HDPE-LDPE blends have more phase inversions, the HDPE-LLDPE butylene blends have a phase inversion between 40 and 60% HDPE content, while the HDPE-LLDPE hexane blends have no or below 40% HDPE content.
- In general, complex viscosity decreases with angular frequency. For HD-LD blends the curves are parallel, while for HD-LLDPE blends are not (they have intersection).
- The complex viscosity shows linear behavior with composition, but some blends have a significant negative deviation. At lower frequency values, complex viscosity is influenced more by angular frequency than at higher frequencies.
- The storage and loss modulus increase in function of angular frequency. The curves of HD-LD blends are parallel, while those of HD-LLDPEs have intersections.
- In the liquid phase, according to rheological measurements, the HDPE-LDPE blends are not or partially miscible, while the HDPE-LLDPE blends are probably miscible.
- The results can be used in industry, for example, in the recycling of mixed polyethylene waste, from which new polyethylene mixtures are made. These mixtures can contribute to the application of covering cables in the automotive industry, where the recycling rate is of particular importance.

## Figures and Tables

**Table 1:** Polymer characteristics of the examined materials.

Name of Raw Material	Investigated Property	
	MFR Values (g/10 min)	Density at 23 °C (kg/m^3^)
HDPE TIPELIN FA 381-10	0.28	937
LDPE TIPOLEN FB 243-51	0.75	921
LLDPE Exceed 3518	3.5	918
LLDPE Flexirine CL10U	2.5	918

## Data Availability

Not applicable.
